# Psychotropic medication profiles and future treatment risk among Northern Irish adults: a Latent Transition Analysis

**DOI:** 10.23889/ijpds.v9i5.2655

**Published:** 2024-09-18

**Authors:** Matthew Wray, Donal McLaughlin, Pratibha Vellanki, Zoe White, Josie Plachta, Leah Maizey

**Affiliations:** 1 Office for National Statistics

The quality of linkage techniques has a direct impact on the quality of statistics produced on the linked data, and as such, ensuring linkage algorithms are of the highest quality is a vital mission of the Office for National Statistics (ONS).

Deterministic hierarchical matchkeys are a linkage technique that applies a list of conditions to classify links, where records can only be linked once. The aim of the hierarchy is to classify correct links by running higher precision matchkeys first, removing linked records from the matching pool, and then running lower quality keys so that they are less likely to make incorrect links. Currently, no optimal method for ordering match-keys exists. Here we present novel research on a generalisable algorithmic approach to finding optimal matchkey orderings.

A deterministic loop over a consistent set of matchkeys was used to link ONS’s 2021 Census to the 2021 Census Coverage Survey (CCS). A range of experimental ordering methods were implemented. Gold standard linked data was used for quality determination to produce metrics such as precision and recall for grading and comparing the experimental ordering methods. The required expertise of the user and computational efficiency were considered when comparing and recommending ordering methods.

Removing historically subjective matchkey ordering methods and replacing with a deterministic ordering algorithm, can lead to better data linkage outcomes with reductions in linkage error. This can provide greater confidence to users of official statistics that linked data underpinning them are more reliable, trustworthy, and meaningful.

